# Climate Change, Habitat Loss, Protected Areas and the Climate Adaptation Potential of Species in Mediterranean Ecosystems Worldwide

**DOI:** 10.1371/journal.pone.0006392

**Published:** 2009-07-29

**Authors:** Kirk R. Klausmeyer, M. Rebecca Shaw

**Affiliations:** The Nature Conservancy, San Francisco, California, United States of America; Umea University, Sweden

## Abstract

Mediterranean climate is found on five continents and supports five global biodiversity hotspots. Based on combined downscaled results from 23 atmosphere-ocean general circulation models (AOGCMs) for three emissions scenarios, we determined the projected spatial shifts in the mediterranean climate extent (MCE) over the next century. Although most AOGCMs project a moderate expansion in the global MCE, regional impacts are large and uneven. The median AOGCM simulation output for the three emissions scenarios project the MCE at the end of the 21^st^ century in Chile will range from 129–153% of its current size, while in Australia, it will contract to only 77–49% of its current size losing an area equivalent to over twice the size of Portugal. Only 4% of the land area within the current MCE worldwide is in protected status (compared to a global average of 12% for all biome types), and, depending on the emissions scenario, only 50–60% of these protected areas are likely to be in the future MCE. To exacerbate the climate impact, nearly one third (29–31%) of the land where the MCE is projected to remain stable has already been converted to human use, limiting the size of the potential climate refuges and diminishing the adaptation potential of native biota. High conversion and low protection in projected stable areas make Australia the highest priority region for investment in climate-adaptation strategies to reduce the threat of climate change to the rich biodiversity of the mediterranean biome.

## Introduction

The mediterranean biome is a global conservation priority [1], [2] owing to high plant species diversity and density that rivals that of tropical rainforests [3], [4]. The biome's mild climate and proximity to the ocean also makes it attractive to humans, resulting in disproportionately high conversion for agriculture, development, and other human uses [5], [6]. Found on five continents, the mediterranean biome includes the Mediterranean Basin, the western United States (California) and Mexico (northwest Baja), central Chile, the cape region of South Africa, and south and southwestern Australia [4]. These five areas cover just 2% of the Earth's land area, but support 20% of the Earth's known vascular plant diversity [3], [7]. Despite this biome's relative biological wealth, formal land management for biodiversity conservation is lagging, as it has the second lowest level of land protection of all the 13 terrestrial biomes [5]. By 2100, the mediterranean biome is projected to experience the largest proportional loss of biodiversity of all terrestrial biomes due to its significant sensitivity to multiple biodiversity threats and interactions among these threats [8].

The mediterranean biome's extraordinary plant diversity and endemism are a result of the evolutionary processes induced by the characteristically unique annual cycles of extended summer drought and cool wet winter, high topographic variation, and low soil fertility [9]. Climate change resulting from increases in atmospheric concentrations of greenhouse gases will impact the extent and distribution of the mediterranean climate, posing a threat to the survival of many species. While biome level analyses are rare, there has been a recent proliferation of climate change impacts studies specific to species and habitats in each of the five mediterranean regions [10]–[14]. These studies generally project significant reductions in endemic species range sizes. For example, in California, 66% of the endemic plant taxa will experience >80% range reductions within a century [14]. Midgley et al. projected a 51–65% reduction in the mediterranean biome in South Africa by 2050, and that only 5% of the endemic Proteaceae species modeled would retain more than two thirds of their current range [15]. However, each of these studies is limited to one of the five mediterranean regions and generally focuses on the results from one to a few of the 23 atmosphere-ocean general circulation models (AOGCMs). In this analysis, we focus on projected shifts in the mediterranean climate using a consistent methodology worldwide. This allows for comparisons between regions and highlights areas that are in most urgent need for climate change adaptation action. We present the first biome-level analysis of global climate change using all AOGCMs analyzed in the Intergovernmental Panel on Climate Change's (IPCC) Fourth Assessment Report [16]. Finally, we estimate the potential for facilitation of species adaptation within a region via the climatic stability of protected areas or via the migration pathways to optimal climatic conditions, based on current distribution of areas managed for biodiversity conservation, current patterns of land conversion, and magnitude of future impacts of climate change. We refer to this measure as *extrinsic adaptation potential* which illuminates characteristics of the landscape that facilitate species adaptation, in contrast to species-specific characteristics that determine *intrinsic adaptation potential* such as dispersal ability or genetic diversity. Intrinsic and extrinsic adaptation potential together defines the adaptation potential of a species.

## Materials and Methods

The mediterranean biome is typically mapped using a combination of climate characteristics and plant assemblages that vary by region. One widely-used delineation of this biome is a collection of ecoregions mapped by the World Wildlife Fund that covers 2.2% of the earth and is based upon climate and plant associations [2]. As the climate changes, the impacts on the climate characteristics across all five mediterranean regions will be mechanistically similar, but the impacts on the plant assemblages will differ as the definition and composition of mediterranean vegetation differs among regions. For this reason, this analysis focuses on the climatic impacts and maps the mediterranean climate extent (MCE) across all five regions based solely on climatic factors and not plant assemblages.

Although there are varying definitions for the mediterranean climate, we chose one definition that can be readily mapped with available climate data and has minimal over prediction into areas that are not part of the mediterranean biome (see supporting information Text S1 for further discussion and sensitivity analysis). According to this definition, published by Aschmann [17], an area is within the MCE if it meets five conditions; 1) The winter must be wetter than the summers (>65% of the precipitation falls in the winter half of the year), 2) the annual precipitation must not be too low (>275 millimeters (mm)), 3) nor too high (<900 mm), 4) the winter must be cool (<15°Celsius (C) mean temperature for the coldest month of the year), but 5) it can not have too much frost (<3% of the annual hours are below freezing). We used the WorldClim [18] high resolution (2.5 arc-minutes or ∼5 kilometer (km) horizontal resolution at the equator) grids of global climate data summaries from 1960–1990 to map the current MCE where all five Aschmann conditions are met (see supporting information Text S1 for more information about the WorldClim dataset).

Data for projections of future climate conditions were derived from the results of the AOGCMs run to support IPCC's Fourth Assessment Report. The data [World Climate Research Programme's (WCRP) Coupled Model Intercomparison Project (CMIP) phase 3 multi-model dataset] include seven future emissions scenarios. Three of these scenarios are used most often by modeling groups and are considered representative of low (B1 or stabilization at 550 ppm atmospheric CO_2_), moderate (A1b or stabilization at 720 ppm atmospheric CO_2_) and high (A2 or no stabilization) emission trajectories [19]. We compiled the AOGCM output data for monthly surface air temperature and precipitation flux for the 20^th^ century and the 21^st^ century for three future emissions scenarios. While some modeling groups have generated multiple simulations for a given scenario and others have done no simulations for a given scenario, we analyzed all available AOGCM simulations in the CMIP multi-model dataset, including 48 low emission simulations, 52 moderate emission simulations, and 36 high emission simulations, for a total of 136 simulations of future climate (see supporting information Text S1 and Table S1). By doing so, we treat each AOGCM simulation for a given emissions scenario as a unique and probable experimental outcome and average the results, thereby elucidating a more robust set of potential climate outcomes.

To reduce the variability associated with annual climate projections, we averaged the monthly data in the AOGCM simulation results to two 30-year periods; one “current” and one “future”. The WorldClim data is primarily derived from 1960–1990 weather records, so we averaged the monthly data from January 1960 to December 1989 for each of the modeled 20^th^ century simulations to generate the current time period. The majority of the model simulations end in 2100, so we averaged the monthly data from January 2070 to December 2099 for each of the modeled 21^st^ century simulations to generate the future time period. We then subtracted the modeled current data from the modeled future data to reduce modeling biases and generate projected climate anomalies. For example, an AOGCM simulation may have modeled the average July temperature for a specific area to be 24°C for the current time period (1960–1989) and 27°C for the future time period (2070–2099), so the projected climate anomaly for that area would be 3°C. We used the change factor approach to downscale the projected climate anomalies from the coarse resolution of the AOGCMs to the finer resolution of the WorldClim data. This method involves interpolating the projected climate anomalies and adding the interpolated data to the current climatology (see supporting information Text S1).

We applied Aschmann's [17] conditions to generate binary maps of the projected future MCE for each AOGCM simulation. The size of the projected future MCE in each region was compared to the size of the current MCE for each AOGCM simulation, and the average and 5, 10, 25, 50, 75, 90, and 95 percentiles of the projected percent change for all simulations in a given emissions scenario were calculated. This provides both a measure of the range and the central tendency of the ensemble of projected changes [20]. We considered using a weighted-average, but since we do not have a testing dataset for the future locations of the MCE, and other studies have found little increases in predictive power with a weighted average compared to a non-weighted average [21], we used only the average.

We spatially combined the current MCE and all of the future MCE projections to calculate the percent of AOGCM simulations predicting an expansion or contraction of the MCE for each grid cell on the globe. We defined seven categories based on whether areas were in the current MCE or not, and the number of AOGCM simulations that project the area will be in the future MCE (Table 1). These categories were mapped using the suite of AOGCM simulations for each emissions scenario.

**Table 1:** Mapped categories for the MCE future projections.

Category	Area in current MCE?	Percent of AOGCM simulations projecting area will be in future MCE
Confident Stable	Yes	90–100%
Likely Stable	Yes	66–90%
Uncertain	Yes	33–66%
Likely Contraction	Yes	10–33%
Confident Contraction	Yes	0–10%
Confident Expansion	No	90–100%
Likely Expansion	No	66–90%

We determined the amount of land protected and modified through development and land conversion within the current and projected future MCE [5]. We used all World Conservation Union categories (I to VI) in the 2006 World Database on Protected Areas to map areas that are protected in the current MCE and in the areas where the MCE is projected to expand (www.unep-wcmc.org/wdpa) [22]. Marine protected areas were not included, and protected areas with only point location were mapped as circles with the correct area. We converted the polygon data to a binary 2.5 minute resolution grid by assigning a grid cell a value of 1 if the center of the cell falls within a protected area polygon, and 0 if not. For spatial data on modified or converted areas, we used the areas classified as “cultivated and managed areas” and “artificial surfaces” in the Global Land Cover 2000 (www-gvm.jrc.it/glc2000) [23]. We converted these data to a binary 2.5 minute resolution grid where 1 indicated a grid cell is converted and 0 indicated it is not. We performed a spatial combination of these two binary grids with the current and projected future MCE grids. Areas that were classified as both protected and converted were considered converted. From this combination of grids, we could determine the percent of the MCE that is protected and converted to human land uses.

## Results

The MCE at the end of the 20^th^ century covered just over 1.5 million km^2^, according to Aschmann's [17] definition. This is a conservative definition of the MCE and reflects the core areas of the mediterranean biome. For comparison, on a commonly used map the mediterranean biome covers 3.2 million km^2^ [2], or over twice the area in the current MCE. Approximately 60% of the current MCE occurs in the Mediterranean Basin, and covers a portion or all of the following countries: Algeria, Cyprus, Greece, Iran, Iraq, Israel, Italy, Jordan, Lebanon, Libya, Malta, Morocco, Portugal, Spain, Syria, Tunisia, and Turkey. The remaining MCE occurs in Australia (25%), the United States/Mexico (9%), Chile/Argentina (4%), and South Africa (2%).

**Figure 1:**
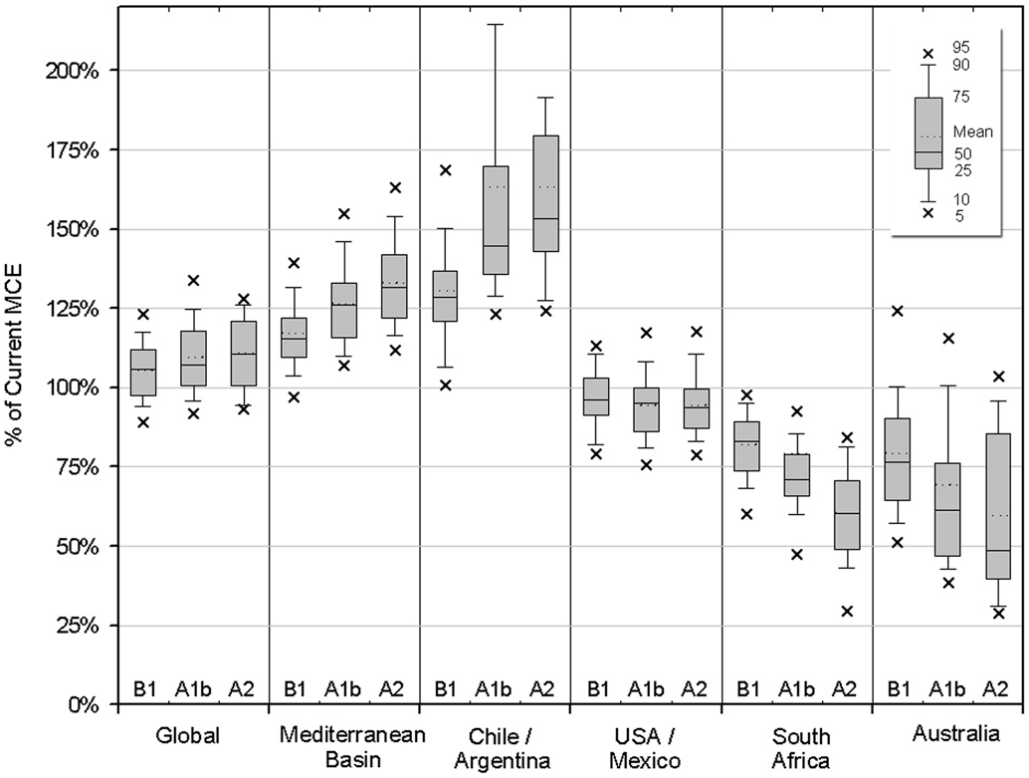
Relative size of the projected future (2070–2099) to the current (1960–1989) MCE. The results are presented in box and whisker diagrams representing the percentiles of the AOGCM simulations for the B1 (low), A1b (medium) and A2 (high) emissions scenarios. The solid line within each box represents the median value, and the dotted line the mean value. The top and bottom of the boxes shows the 75th and 25th percentiles, the top and bottom of each whisker shows the 90th and 10th percentiles, and the small X's show the 95th and 5th percentiles. The left-most portion of the figure represents the results for all five regions, and the region specific results are presented to the right. The 95th percentile values for Chile/Argentina for the moderate and high emissions scenarios (320% and 235%) are not included to show more detail in the remaining regions.

The majority of AOGCM and emissions scenario projections of the MCE at the end of the 21^st^ century (or future MCE) are larger than the MCE at the end of the 20^th^ century (or the current MCE). The median future MCE increases to 106, 107, or 111% of its current size, for the low, medium, and high emissions scenarios, respectively (Figure 1). However, this pattern is not consistent within each region. Instead, there is a disparity between the regions with some projected to experience an increase in the MCE in the future and some projected to experience a decrease in the MCE. Almost all of the AOGCM simulations project an increase in the MCE in the Mediterranean Basin with the median future MCE increasing to 115, 126, or 132% of its current size for the low, medium, or high emissions scenarios, respectively. The median projected increases are greater in Chile/Argentina, ranging from 129% for the low to 153% for the high emissions scenario. In the United States/Mexico region, the projected change in the MCE is less dramatic, with the median future MCE decreasing to 96, 95, and 94% of its current size, for the low, medium, and high emissions scenarios, respectively. In South Africa, greater than 90% of the AOGCM simulations project a decrease in the future MCE, with the median estimates ranging from 83% of the current MCE for the low to 60% for the high emissions scenario. In Australia, the projected area reduction is more extreme, with median estimates ranging from 77% of the current MCE for the low to 49% for the high emissions scenario.

**Figure 2:**
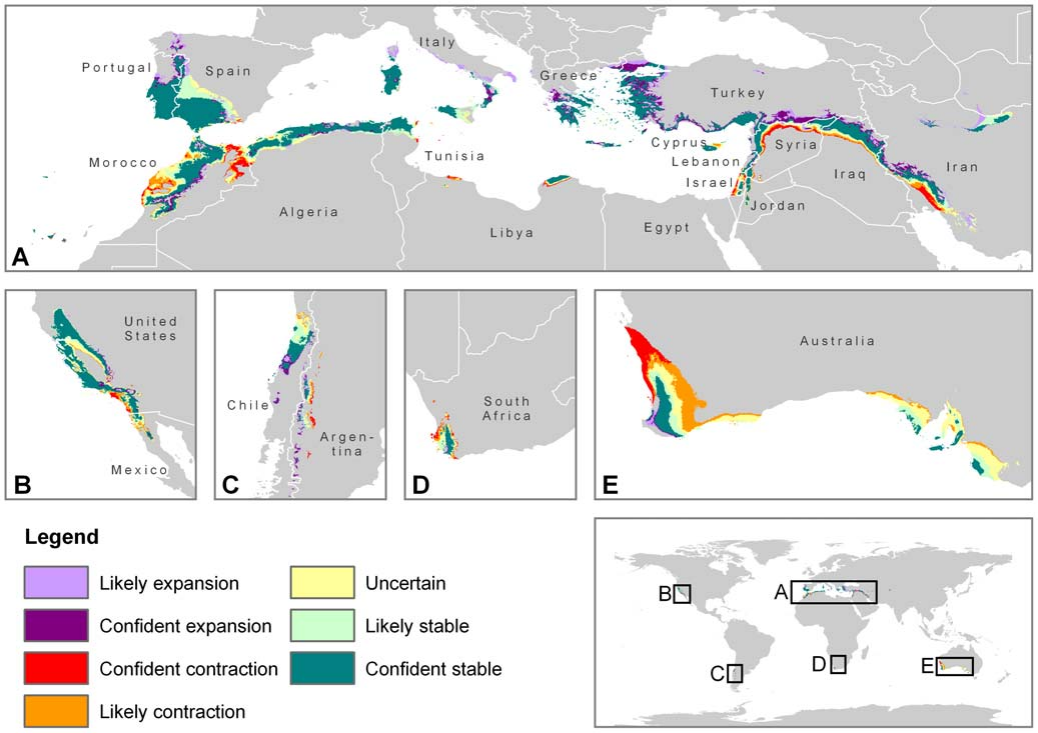
Projected status of the MCE in 2070–2099 relative to 1960–1989 under high (A2) emissions scenario. The projected status is considered likely if at least 66% of the AOGCM simulations agree, confident if at least 90% agree. Maps A. through E. are un-projected at 1∶50,000,000 scale.

By overlaying all of the future MCE projections, we were able to map all of the grid cells on the globe where the MCE is likely to expand, contract, and remain stable with varying levels of confidence (Figure 2). We show that there are areas of contraction even within regions that are projected to have a net increase in the MCE. For example, the median projection of the future MCE in the Mediterranean Basin is larger than the current MCE, but most AOGCM simulations project contractions in Morocco and in the Middle East. The geographic separation between the areas of contraction and expansion within each region highlighted in Figure 2 will have important implications for adaptation of native plants and animals with limited dispersal or migration capabilities.

In addition to showing where the MCE is projected to contract, we also wanted to determine if there was AOGCM agreement on why it will contract (e.g., a warmer winter temperature, or less annual precipitation). These changes will have important implications for the persistence of native plants and animals in the mediterranean biome. To do this, we determined the level of agreement among the AOGCM simulations on which Aschmann's conditions were no longer met for the areas with a projected contraction. We report the results for areas where at least 90% of the AOGCM simulations agree that the MCE will contract *and* agree on the changing climatic condition causing the contraction under the high emissions scenario. Across all five regions, we can project with the most confidence that 7.2% of the current MCE will contract. Over half of this projected contraction, or 4.0% of the current MCE, results from warming in the winter. Almost a quarter of the projected contraction, or 1.7% of the current MCE, results from a drop in total annual precipitation below the 275 mm threshold. For one fifth of the projected contraction, or 1.5% of the current MCE, the AOGCM simulations agree that an area will contract, but they do not agree on which condition will change (supporting information Table S2). By country, most of the loss in Australia, the United States, Iran, Israel, and Libya is attributable to a warming winter, while the majority of the loss in Argentina, South Africa, Morocco, and Syria is due to a drop in annual precipitation.

**Figure 3:**
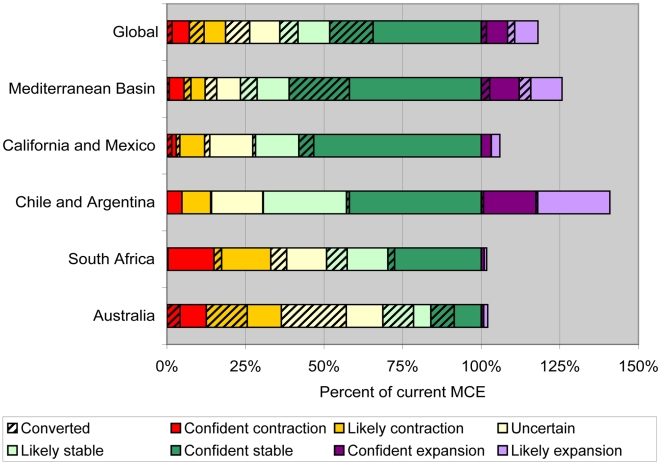
Projected future changes and current conversion status of the MCE under the high (A2) emissions scenario. The percentages indicate the portion of the area within the current MCE.

Current land conversion and protection status and configuration relative to these climatic changes will play an important role in determining the extrinsic adaptation potential for the species of the mediterranean biome. Approximately one third of the area in the current MCE has already been converted to agricultural and urban land uses. If most of the converted land is in areas where the MCE is projected to contract, extrinsic adaptation potential will not be significantly reduced because these areas are poor habitat for native species. However, if the areas projected to have a stable or expanded MCE are disproportionately converted, this will exacerbate the negative impacts of climate change on biodiversity in the mediterranean biome. When looking across all five regions, we found the land conversion patterns are similar in areas where the MCE is projected to contract with confidence (23%) and in areas projected to remain stable with confidence (29%), but the regional patterns were more variable (Figure 3). In California and Mexico, extrinsic adaptation potential is conserved in the future because most of the conversion lies in areas that are projected to contract, and there is little conversion in the areas of stability. Similarly, Chile/Argentina and South Africa also have low levels of conversion in the confident stable areas. The opposite is true in Australia, where 64% of the likely stable area and 49% of the confident stable area is already converted, greatly diminishing the extrinsic adaptation potential of native biota.

**Figure 4:**
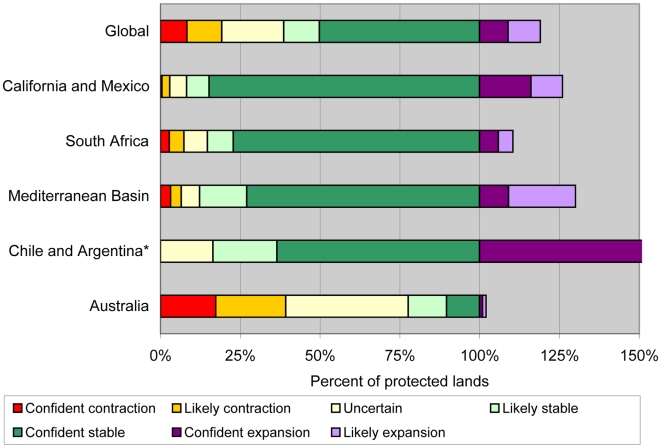
Projected future status of the MCE on protected lands under the high (A2) emissions scenario. The percentages indicate the portion of the current protected lands within the current MCE. There is over 5 times the amount of protected area in the confident and likely expansion area in Chile and Argentina relative to the amount in the current MCE, but the full extent is not shown in the chart to show more detail in the other regions.

At 4%, the level of protection for biodiversity in the current MCE is below that of the more expansive mediterranean biome (5%) and well below the global average (12%) for all terrestrial biome types [5]. We wanted to determine if the level of protection is higher or lower in areas with high likelihood of retaining a mediterranean climate. For the entire biome, just over half of the existing protected areas are projected to retain the mediterranean climate with high confidence, even under the high emissions scenario (Figure 4). The projected status of protected lands in some regions is much better, as over 70% of the protected areas in California/Mexico, South Africa, and the Mediterranean Basin are in the confident stable areas. In stark contrast, the MCE is projected to contract or is uncertain in over 75% of the protected areas with mediterranean climate in Australia, and there is almost no projected expansion into other protected areas to offset this loss.

## Discussion

This analysis provides the most comprehensive assessment to date of how climate change is projected to impact mediterranean climates in all five mediterranean regions across the globe and the implication of the climate change for adaptation potential of the native biota. By analyzing the full suite of multiple simulations of 23 AOGCMs under multiple emissions scenarios, we are able to present quantitative estimates of the level of agreement in the projected contractions in the MCE. Previous studies focus on one or several “bookend” AOGCM runs that attempt to encompass the range of variability in the future projections. If one model projects a wetter future and the other a drier future, the recommendations for conservation action could be vastly different with no method for determining which future is more likely. This study quantifies the level of agreement between a full suite of projected climatic futures, providing a more robust and conservative estimate of impact and thus more confidence to support conservation action.

**Figure 5:**
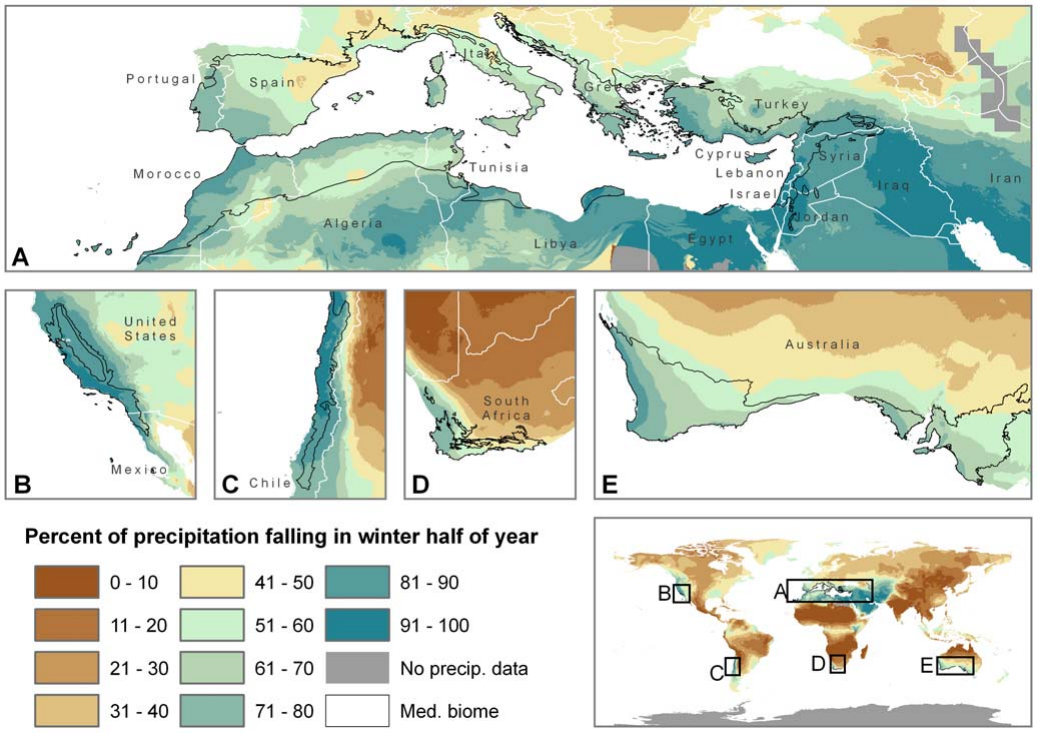
Percentage of precipitation falling in the winter half of the year from the WorldClim dataset. The depiction of the mediterranean (med.) biome is from Olson et al. [2] and is provided to show areas that are often considered part of the mediterranean biome but have less that 65% of the precipitation falling in the winter half of the year, and thus are not included in the current MCE.

One of the first challenges to projecting the impacts of climate change on mediterranean ecosystems worldwide is defining the MCE. The extent of the mediterranean *biome* is typically mapped based on a combination of climate characteristics and plant assemblages that vary from region to region [2]. Projecting how plant assemblages will shift in response to climate change is subject to significant uncertainty because it requires compounding the uncertainty with projecting climate change with the uncertainty inherent in projecting future distributions of individual species [24], [25]. In this analysis, we minimize the uncertainty and focus on mediterranean *climate* shifts in the future. As such, we utilize a conservative definition of mediterranean climate [17] that is consistent across all mediterranean regions and minimizes “false-positives” (areas that are not considered part of the mediterranean biome). Some areas that are traditionally considered part of the mediterranean biome are not within our current or future MCE, including the south coast of France, western Italy, northeastern Spain, portions of central Chile, and the southern coast of South Africa. Most of these areas receive less than 65% of their rain in the winter, and thus do not meet the first Aschmann condition (Figure 5). Despite the conservative nature of this definition, we do include some false-positives, including parts of Argentina and the Middle East. These commissions could be the result of a lack of climate station data in these more remote and mountainous areas. We performed a sensitivity analysis with the Köppen definition of mediterranean climate, which is less conservative (Figure S1). This definition identifies more of the traditional areas, but also includes large areas of false-positives. However, when focusing in on the five mediterranean regions, the results of the projected fate of the MCE are very similar using both definitions (see the full analysis in supporting information Text S1)

While there are some significant discrepancies between our map of the current MCE and commonly used maps of the mediterranean biome, such as the one mapped by Olson et al. [2], preliminary analysis indicates significant overlap between the current MCE and the mapped hotspots of plant richness and endemism within the mediterranean biome. Our current MCE corresponds well with the biogegraphical sectors with high incidences of plant endemism in the Mediterranean Basin [7], areas of high modeled endemic *Banksia* species richness in western Australia [11], and areas of high modeled endemic plant richness in the California Floristic province [14]. In South Africa, an analysis of both modeled and observed plant richness shows the area identified in the current MCE in the Western Cape as a hotspot [26]. This correlation between areas of high plant richness and the MCE does not diminish the need for conservation action in all areas of the mediterranean biome, but it does provide support for the theory that the core mediterranean climate is an important driver of plant endemism and diversity, and that changes in climate could threaten the survival of these endemic plants.

Across the entire mediterranean biome, most of the 23 AOGCMs project a minor increase in the MCE, with significantly large increases in some regions, and significant decreases in others. Not surprisingly, the physical characteristics in each region help to explain some of the disparity between areas of contraction and expansion. High topographic diversity and contiguous land toward the nearest pole provide room for the expansion of the MCE in the United States, Chile, Argentina, Greece, Turkey, Spain and Portugal. In South Africa, there is topographic relief so the MCE can retreat upslope into the Western Cape Fold Mountains, but there is no contiguous land toward the south pole cutting off future expansion of the MCE. Similarly in Morocco, the Atlas Mountains provide topographic diversity, but the Mediterranean Sea blocks expansion toward the north. Southwestern Australia is a flat highly weathered plateau [27] and is contained by the Indian Ocean to the south, resulting in the largest projected contraction of all the regions.

While this is the first global assessment of the impacts of climate change on the MCE, our results are consistent with other regional biome and species level analyses. The IPCC determined that the mediterranean biome as a whole is threatened by desertification from expansion of semi-arid and arid system under relatively minor warming and drying scenarios, and projects significant regional vegetation and species range shifts [28]. In South Africa, despite differences in the current spatial extent, Midgley et al. projected areas of future mediterranean biome contraction and stability in similar areas as our analysis [15]. Similarly, Williams et al. and Hannah et al. found the higher elevation areas of the Western Cape in South Africa support high levels of endemic Proteaceae species richness, and will be important habitat for dispersal by 2050, while the low-lying areas north of Cape Town are high in richness now, but are not protected to support the species in the future [29], [30]. These areas correspond well with our projected areas of higher elevation stability and lower elevation contraction in South Africa. Fitzpatrick et al. studied potential range shifts for native *Banksia* species in Western Australia and found the areas of greatest percent loss in richness in the arid interior, while the projected loss was less severe in the coastal areas and Fitz-Sterling Ranges [11]. These results are consistent with the results presented in Figure 2, although Fitzpatrick et al. did project species richness increases along the western coast of Australia, while our analysis found almost no projected expansion of the MCE in Australia. Loarie et al. identified areas of future refugia for species with projected range reductions in the mountainous areas along the central coast and foothills of California, USA and Baja California, Mexico [14], which correspond well with our areas of projected MCE stability. Benito-Garzón et al. studied a series of tree species in the Iberian Peninsula and found that some of the mediterranean species had the least projected range reductions and could potentially expand north and into higher elevations [13], which is consistent with our findings in Spain and Portugal. In general, the results from these biome and species level analyses that focused on one region at a time support our findings as well as the urgent need for conservation action to increase the extrinsic adaptation potential of the native biota of the mediterranean biome.

This analysis shows that climate change puts areas with the some of the highest levels of plant diversity and endemism on the Earth at risk. As shown in Figure 2, the Mediterranean Basin, Morocco and Israel contain large areas of projected contraction with no adjacent areas of expansion. The mediterranean portions of Morocco contain almost 13 plant species per 1000 km^2^, while Israel contains 200 [7]. The cape region in South Africa and southwest Australia are the other two regions with large projected losses, and they contain 95.5 and 71 plant species per 1000 km^2^, respectively [7]. For comparison, there is 1 plant species/1000 km^2^ in Europe, 6.5 in Brazil, and 40 in Columbia [7]. The current MCE in these four threatened countries contain 22,400 plants species, 12,925 of which are found no where else [7].

The adaptation potential of the species native to the mediterranean biome will be further limited beyond the direct impacts of climate change analyzed here. Native plant species in all mediterranean regions, except perhaps Chile, are well adapted to natural fire regimes, but a hotter and drier climate has been observed to promote [31] and is projected to promote significant alterations to the fire regime beyond those created by decades of human fire management [28], [32], [33]. While rising atmospheric CO_2_ levels could provide benefits to mediterranean plant species [34], the effects are altered when multiple factors of change are considered, including fire, drought, temperature increase, nitrogen deposition, and invasive species [35]–[38]. The rich plant diversity of mediterranean systems is explained in part by the plant adaptations to survive in low nutrient soils, such as California's serpentine soils, Australia's kwongan, and South Africa's fynbos [3], [4], [9]. The patchy nature of soils will act as a barrier and will make species migration in response to a changing climate more difficult. The intrinsic adaptation potential of some mediterranean endemics, particularly in South Africa and Australia, is limited by the relatively short seed dispersal distances and lack of colonization ability of these plants [11], [29], [39], [40]. While these indirect and interacting impacts of climate change are not explicitly considered in this analysis, they are likely to further limit the adaptation potential of mediterranean species.

Despite the significant projected contractions in the MCE, this analysis does offer some reasons for hope and some guidance to direct future conservation action. Approximately 50% of the biome is projected to remain stable with confidence, even under the high emissions scenario. Establishment and management of protected areas in these areas in all five regions represent sound investments given our current understanding of future change, and will help to secure future refugia for endemic species from other threats such as land conversion. However, given the uncertainties associated with the indirect effects of climate change, a conservative conservation approach should also include gene banking and ex-situ conservation for the rich floras of highly threatened regions like Israel, Morocco, South Africa, and Australia.

Since this analysis was conducted using a consistent methodology across all five regions, we can use the results to determine the highest priority regions for action. As shown in Figure 3, South Africa and Australia have the large projected contractions in the MCE. The existing protected area network covers almost 7% of the current MCE in these two regions. In South Africa, the protected areas are concentrated in the higher elevations where the MCE is projected to remain stable, so 77% of the current protected areas are projected to retain mediterranean climate and only 3% are projected to have a different climate in the future with high confidence, even under the high emissions scenario. In contrast, the protected areas in southwest Australia are concentrated in the drier inland portions of the MCE, so only 10% of the current protected areas will remain stable while 17% will likely shift to a new climate. These results suggest that some of the first strategies to enhance the extrinsic adaptation potential and to reduce the threat of climate change to the rich biodiversity of the mediterranean biome might include establishing new protected areas within areas projected to remain stable, improving land management, and restoring native habitat in southwest Australia. In particular, restoration efforts could focus on creating corridors or stepping stones of native habitat to connect isolated remnant vegetation patches in the areas with projected MCE contraction to the native habitat in the areas projected to remain stable. While this strategy has been advocated before [41], [42], and is currently underway in projects like the Gondwana Link (www.gondwanalink.org), this analysis highlights specific areas where this strategy could be implemented to improve the extrinsic adaptation potential of native species in the face of climate change.

Climate observations over the past century indicate the climate in the mediterranean biome is changing [43]. There is also a high level of agreement in the AOGCM simulations that most of the biome will continue to get hotter and drier. This study shows where there is agreement that the biome will shift, how the threats to biodiversity from these shifts will be exacerbated by current land use and land protection patterns, and highlights which regions are in need of the most urgent conservation attention. This conservation attention is required to establish protected areas and connectivity pathways to ensure the current investment in protected areas remains secure and facilitates and enhances the extrinsic adaptation potential of the species of the mediterranean biome.

## Supporting Information

Text S1 Supporting information text
(0.11 MB DOC)

Table S1 AOGCM simulations downscaled and analyzed. Table 8.1 in Chapter 8 of the IPCC's Fourth Assessment Report contains more information about these models and the references for the ocean, atmosphere and coupling components.
(0.05 MB DOC)

Table S2 Reasons for MCE contraction for the high emissions scenario (A2) where 90–100% of the AOGCM simulations agree. Unless noted, figures are percent of current MCE in each region or for all regions.
(0.04 MB DOC)

Figure S1 Projected status of the MCE using the Köppen definition in 2070–2099 relative to 1960–1989 under high (A2) emissions scenario. The projected status is considered likely if at least 66% of the AOGCM simulations agree, confident if at least 90% agree. Maps A. through E. are un-projected at 1∶50,000,000 scale.
(0.63 MB TIF)
