# Supplementary material for: Climate Change, Habitat Loss, Protected Areas and the Climate Adaptation Potential of Species in Mediterranean Ecosystems Worldwide
Source: PLoS One. 2009 Jul 29;4(7):e6392. doi: 10.1371/journal.pone.0006392 (PMC2712077; doi:10.1371/journal.pone.0006392)
Supplement: Text S1 — Supporting information text (0.11 MB DOC) [file pone.0006392.s001.doc]

**Supporting Information Text S1**

*Mapping Current Mediterranean Climate*

Several global and regional climate studies have developed criteria for defining the mediterranean climate extent (MCE). These studies agree on the presence of a dry period within the hot season as part of the definition, but disagree on the specifics [1,2]. An early global climate zone study that identifies a mediterranean zone and is still used today is the Köppen classification [3-7]. However, the cold boundary for the Köppen mediterranean climate is too low and thus includes broad-leafed deciduous and dense coniferous forests that are not characteristic of the mediterranean biome [8]. As a result, it overestimates the extent of mediterranean climate and includes areas that are not traditionally considered mediterranean (central Mexico, the Democratic Republic of Congo, and northern India) (see overview map in Figure S1). Despite these limitations, the Köppen classification can be mapped with available climate layers, so we used it to test how sensitive our results are to the definition of mediterranean climate (see methods and results of this sensitivity test below). Other analyses have defined gradients within the MCE by comparing the minimum monthly temperature and an index of aridity [9,10]. However, these studies do not provide the boundary conditions necessary to map the MCE at a global scale.

For this analysis, we focus on the definition developed by Aschmann [8] because it can be readily mapped with existing global climate data, it has been used in other studies [2,11], and when mapped, it corresponds well with other mapping efforts of the mediterranean biome [12]. While this definition is somewhat conservative and does not include certain areas traditionally considered mediterranean, it does reflect the core areas of the mediterranean biome.

To map the current MCE, we used the high-resolution WorldClim dataset, version 1.4 ([www.worldclim.org](http://www.worldclim.org/)). These data correspond to the average climate conditions between ~1950 and 2000, with most of the data from 1960-1990 records. We used the 2.5 minute resolution (~5 kilometer (km) horizontal resolution at the equator) ESRI format grids of monthly total precipitation in millimeters (mm) and monthly maximum and minimum temperature in degrees Celsius (°C). Although higher resolution WorldClim grids are available (0.5 minute resolution), we did not use them because the 2.5 minute resolution data are appropriate for the biome scale of the analysis. WorldClim data are available for all land areas of the globe (excluding Antarctica). The uncertainty associated with these data is estimated to be highest in areas with mountains and few weather stations [13]. With the exception of western Argentina and portions of Spain, most of the areas in the MCE have a high density of weather stations. The uncertainty of the current extent in the MCE is going to be higher in areas with greater topographic diversity. While higher resolution regional climate datasets are available for many of the regions (e.g., PRISM in the United States), we wanted to use one global dataset to facilitate cross regional comparison.

All of the data storage, management, and analysis of the WorldClim data were completed with ESRI ArcGIS 9.1 software ([www.esri.com](http://www.esri.com/)). The Aschmann mediterranean climate definition requires several derivative metrics not provided in the WorldClim dataset. To generate monthly mean temperature grids, we added the monthly maximum and minimum grids and divided by two. We also generated the total annual precipitation (psum), the total precipitation in the winter half of the year (November to April in the northern hemisphere and May to October in the southern hemisphere, p_wsum_asch), the average annual temperature (tave), the range in monthly temperatures (trange), and the minimum mean monthly temperature (tmin). The five Aschmann mediterranean climate conditions were calculated as five binary raster grids with a value of 1 if the following conditions were met, and zero otherwise:

1. p_wsum_asch >= (0.65 * psum)
2. psum >= 275
3. psum <= 900
4. tmin < 15
5. tave >= 0.7 * trange + 2.76 [14]

To highlight the areas where all five conditions were met, the five binary grids were multiplied together to result in a final binary grid where 1 meets the Aschmann mediterranean climate definition and 0 does not.

*Spatial Downscaling*

The spatial resolutions of the current generation of global climate models range from 1.1° to 5° (~125 km to ~550 km horizontal resolution at the equator) [15]. While these resolutions are useful for global and continental scale analyses, it is too coarse to see patterns of change at a regional or biome scale. We therefore downscaled the AOGCM outputs to analyze the data with the resolution of the current climate data (~5 km), which is sufficient to see regional patterns of change.

There are various methods of downscaling AOGCM data, including dynamic downscaling, statistical downscaling, and the change factor approach. Dynamic downscaling uses regional climate models that originated from numerical weather prediction. Most regional climate models generate results at a scale of 50 km, although some generate results at 10 km and finer scales [16,17]. As their name implies, they are typically run for a region of the globe, using AOGCM outputs as boundary conditions. A regional climate simulation is time and computer intensive [16], so generating outputs for all five mediterranean regions for 136 AOGCM simulations was not feasible.

Statistical downscaling uses various methods to estimate a relationship between large scale climate variables (“predictors”) and finer scale regional or local variables (“predictands”). This relationship is derived from an observed period of climate and then applied to the output from AOGCMs for future projections. This method is also used for temporal downscaling to project daily or hourly variables, typically for hydrologic analyses [18]. Due to the complexity of determining a significant relationship between the “predictors” and “predictands”, most studies that use statistical downscaling only use the results from one [19-21] or several AOGCMs [22]. We are unaware of studies that have used statistical downscaling for over 100 simulations at a global scale.

For our analysis we used the change factor downscaling approach which involves subtracting or dividing the projected future climate from the 20^th^ century run at the native coarse resolution of the AOGCM. These projected climate “anomalies” are then interpolated to create a seamless surface of projected change at a finer resolution. These interpolated data are then added to the current climate to provide an estimate of future climate. Researchers use the change factor approach when a rapid assessment of multiple AOGCMs and emissions scenarios are required [18,23-26]. We chose this method to ensure we could include results from all available AOGCM simulations. We also performed a retrospective analysis to determine the relative size of the errors associated with this method (see *Change Factor Downscaling Errors* below).

*Change Factor Downscaling*

To conduct the change factor spatial downscaling on the AOGCM data, we generated modeled climate anomalies by subtracting the projected 2070-2099 averaged monthly temperature and precipitation data from the 20^th^ century 1960-1989 averaged monthly data [27]. The tabular data were then converted to ArcGIS point shapefiles, with one shapefile for each of the 136 model simulations. After several tests, we found that the interpolation requires the most computer processing time and a significant amount of computer storage space for the outputs. Increasing the interpolation resolution from 2.5 minutes to 6 minutes cut these requirements by a factor of 10. Using the same methods described in the Supporting Text below, we tested the results from an interpolation to 6 minutes and 2.5 minutes, and found only minor differences in the standard deviation of the errors. Thus, the point data were interpolated to a 6 minute resolution grid using the best interpolation method identified through our retrospective tests (regularized spline method with a factor of 0.1 and a neighborhood of 12 points, see *Change Factor Downscaling Errors* below). Since each grid contains anomaly data for one month and one variable, this generated 3,264 anomaly grids (136 AOGCM simulations * 12 months * 2 variables). These grids were then converted back to the 2.5 minute resolution without interpolation and added to the current climate data to provide global downscaled projections of future climate.

*Change Factor Downscaling Errors*

To ensure errors associated with using the change factor method are within an acceptable range given other errors in the analyses, we conducted a retrospective analysis with historical climate data for the continental United States. The data are from the Parameter-elevation Regressions on Independent Slopes Model (PRISM, <http://prism.oregonstate.edu/>) and include grids of monthly temperature and precipitation data at 2.5 minute resolution. We chose two five-year time periods (1951-1955 and 2001-2005) and averaged the annual temperature and the total precipitation to remove some of the annual variability. We used the 1951-55 as the “base” time period and the 2001-2005 as the “future” time period.

To simulate AOGCM outputs, we generated grids for three representative resolutions of the current generation of AOGCMs; 5° horizontal resolution (~550 km at the equator), 2.8° (~315 km), and 1.4° (~157 km). We then averaged the “future” or 2001-2005 PRISM data within each grid and associated the averages with center points. This is equivalent to an AOGCM that produces accurate results, but is not able to replicate the fine-scale climate data. We also averaged the base or 1951-1955 time period data within each AOGCM resolution grid, and then subtracted the base averages from the future averages. This generates climate anomalies between the two time periods.

To replicate the change factor approach, we used the anomaly data from the center points at the three AOGCM resolutions to interpolate a surface at the PRISM data resolution, and then added this anomaly surface to the base 1951-1955 PRISM data (we converted any negative precipitation values to zero). This created a grid of estimated 2001-2005 climate data to which we compared with the grid of observed 2001-2005 climate data. We tested three common interpolation methods (inverse distance weighting, spline, and kriging) that are available with the spatial analyst extension in ArcMap version 9.1. For each method, we tested various numbers of points included in interpolation (4, 8, and 12 points). For the spline method, we tested the tension and regularized method with weights of 0, 0.1, and 0.5. We were unable to test all combinations of the methods and variables, but we assessed the results iteratively and chose methods with the lowest root mean square errors.

We calculated the mean and standard deviation of the errors for each interpolation method and each AOGCM resolution. As expected, the standard deviation of the error goes up as the AOGCM resolution gets coarser. We averaged the means and standard deviations for the three AOGCM resolutions to compare the various interpolation methods. The regularized spline method with 12 points and a weight of 0.1 had the lowest standard error for temperature of all the methods we analyzed. The same method with 4 points performed slightly better for precipitation, but considerably worse for temperature, so we used the 12 point method. Across the three AOGCM resolutions we tested with this method, the mean temperature error was 0.0023°C and the mean precipitation error was 0.09 mm, indicating a slight positive bias associated with the method in this test. The standard deviation of the error (SDE) was 0.46°C for the temperature data and 73 mm for precipitation. Since the errors are approximately normally distributed, the estimated temperature value in 68% of the grid cells is ± 1 * SDE (±0.46°C) of the actual value, and the estimated temperature value in 95% of the grid cells is ± 1.96 * SDE (±0.90°C) of its actual value. Similarly, the estimated precipitation value in 68% of the grid cells is ±73 mm of the actual value, and the estimated precipitation value in 95% of the grid cells is ±143 mm of its actual value.

In order to determine the significance of these error ranges, we can compare them with the magnitude and uncertainties associated with future climate change projections. Using the full ensemble of AOGCMs, the IPCC estimates the projected increase in the global surface air temperature will be 1.8°C, 2.8°C, and 3.4°C in the low, moderate, and high scenarios, respectively, by 2090-2099 relative to 1980-1999 [28]. For at least 95% of the grid cells, the errors associated with downscaling are less than half of the projected increase in temperatures, so the errors associated with the downscaling method are not likely to mask the warming signal. The IPCC also estimates the future increase in global mean temperature is likely (>66% probability of occurrence) to fall within -40% and +60% of the multi-model AOGCM mean warming projected for each scenario [28]. The errors associated with the change factor downscaling at the likely or 66% confidence level are approximately ±26%, ±16%, and ±14% of the projected temperature increase for the low, moderate, and high emission scenarios, respectively. Thus, the errors introduced when we downscale the data using the change factor method are likely to be less than the errors associated with the AOGCM projections.

There is considerably more spatial and temporal variation and uncertainty in the projected precipitation changes over the next century. The mean estimate from the IPCC multi-model ensemble for the moderate emission scenario indicates a decrease in annual precipitation for most of the mediterranean areas on the order of 40 to 180 mm per year. While the IPCC does not provide uncertainty ranges for these estimates, it does indicate that at least 80% of the models agree on the direction (increase or decrease) of the projected precipitation change in the mediterranean regions, except for parts of California [28]. The errors from downscaling (±143 mm at the 95% confidence level) are large enough to obscure the mean predicted change in precipitation, but until there is more analysis of uncertainty in precipitation models, it is not possible to compare the downscaling errors with the uncertainty ranges associated with modeling future changes in precipitation.

*Sensitivity Analysis with the Köppen Classification*

*Methods*

The Cs climate type in the Köppen classification is often considered a mediterranean climate. The Köppen definition has been modified over time, but a recent study used the following conditions for the delineation of the Cs climate type [5]:

1. The precipitation in the wettest month of the winter half of the year (p_wmax) is greater than 3 times the precipitation in the driest month of the summer half of the year (p_smin)
2. The precipitation in the driest month of the winter half of the year (p_wmin) is greater than the precipitation in the driest month of the summer half of the year (p_smin)
3. The annual precipitation (psum) is greater than a variable threshold value for arid climates (p_th)
4. The precipitation in the driest month of the summer half of the year (p_smin) is less than 40mm
5. The minimum monthly temperature (tmin) is less than 18°C
6. The minimum monthly temperature (tmin) is greater than -3°C
7. The maximum monthly temperature (tmax) is greater than 10°C

The winter half of the year is October-March in the northern hemisphere and April-September in the southern hemisphere, and vice-versa for the summer half of the year. The arid precipitation threshold is dependent on the annual temperature and varies based on the total amount of precipitation that occurs in the winter (p_wsum) and summer (p_ssum) half of the year:

If p_ssum >= .666 * psum, p_th = 2 * tave + 28,

If p_wsum >= .666 * psum, p_th = 2 * tave,

Otherwise, p_th = 2 * tave + 14

The seven Köppen conditions were mapped as binary grid with 1 if the condition was met and zero otherwise using the following equations:

1. p_wmax > 3 * p_smin
2. p_wmin > p_smin
3. psum > p_th
4. p_smin < 40
5. tmin < 18
6. tmin > -3
7. tmax > 10

The binary grid of the extent of mediterranean climate according to the Köppen definition was mapped by multiplying the seven condition grids. This process was done based on the “current” WorldClim climate data and each of the future climate scenarios. Using the same methods as with the Aschmann climate definition, we combined the historical MCE with the future MCE and classified the result according to the projected fate of the MCE and the level of agreement between future projections.

*Results*

The results of the projected fate of the MCE using the Köppen definition are presented in Figure S1. Despite using different sources of climate data, our current map of mediterranean climate corresponds well with the map developed by Kottek et al. [5]. The Köppen definition of the MCE is more inclusive than the Aschmann definition, and thus includes areas that are traditionally considered part of the mediterranean biome but are not part of the Aschmann MCE, including the southern coast of France, the western coast of Italy, and parts of Albania and Croatia (see Figure S1). However, as mentioned above, the current Köppen MCE also includes large areas that are not traditionally considered mediterranean (central Mexico, the Democratic Republic of Congo, and northern India). When combined with the future projections of the Köppen MCE, these large areas are classified as “confident contract.” As a result, when comparing the global results of the fate of the Köppen and Aschmann MCE, the Köppen analysis projected much larger declines in the MCE, even though much of the decline is in areas that are not mediterranean. Instead of focusing on these misleading global results, we present a more qualitative comparison by focusing on results of the 5 regions traditionally considered mediterranean.

The spatial patterns of contraction and expansion are similar in Australia, South Africa, and Chile/Argentina between both the Aschmann definition (Figure 2) and the Köppen definition (Figure S1). In the USA/Mexico, the patterns of contraction are similar with both definitions, but the Köppen definition projects more expansion north and east. The spatial patterns in the Mediterranean Basin are also similar, although there is more projected contraction in Algeria, Spain, and Turkey in the Köppen definition. Despite these differences, the primary conclusions reached when using the Aschmann definition of the MCE hold when using the Köppen definition; areas with more topographic complexity and room for poleward expansion are more likely to have a stable MCE, and Australia is the most at risk from projected contractions.

**Supporting References and Notes**

1. LeHouerou HN (2004) An Agro-Bioclimatic Classification of Arid and Semiarid Lands in the Isoclimatic Mediterranean Zones. Arid Land Research and Management 18: 301-346.

2. Quezel P, Barbero M (1982) Definition and characterization of Mediterranean-type ecosystems. Ecologia Mediterranea 8: 15-29.

3. Köppen W (1900) Versuch einer Klassifikation der Klimate, vorzugsweise nach ihren Beziehungen zur Pflanzenwelt. Geogr Zeitschr 6: 593-611, 657-679.

4. Kalvova J, Halenka T, Bezpalcova K, Nemesova I (2003) Koppen climate types in observed and simulated climates. Studia Geophysica Et Geodaetica 47: 185-202.

5. Kottek M, Grieser J, Beck C, Rudolf B, Rubel F (2006) World map of the Koppen-Geiger climate classification updated. Meteorologische Zeitschrift 15: 259-263.

6. Beck C, Grieser J, Kottek M, Rubel F, Rudolf B (2006) Characterizing Global Climate Change by Means of Koppen Climate Classification. Deutscher Wetterdienst. pp. 139-149.

7. Gnanadesikan A, Stouffer RJ (2006) Diagnosing atmosphere-ocean general circulation model errors relevant to the terrestrial biosphere using the Koppen climate classification. Geophysical Research Letters 33.

8. Aschmann H (1973) Distribution and Peculiarity of Mediterranean Ecosystems. In: Di Castri F, Mooney HA, editors. Mediterranean type ecosystems; origin and structure. Berlin, New York: Springer-Verlag. pp. xii, 405 p. illus. 425 cm.

9. Emberger L (1930) La vegetation de la region mediterraneenne. Essai d'une classification des groupements vegetaux. Rev Gen Bot 42: 705-721.

10. Nahal I (1981) The Mediterranean Climate from a Biological Viewpoint. In: Di Castri F, Goodall DW, Specht RL, editors. Mediterranean-type shrublands. Amsterdam; New York: Elsevier Scientific Pub. Co.: distributors for the United States and Canada, Elsevier North-Holland. pp. xii, 643 p.

11. Di Castri F, Goodall DW, Specht RL (1981) Mediterranean-type shrublands. Amsterdam; New York: Elsevier Scientific Pub. Co.: distributors for the United States and Canada, Elsevier North-Holland. 643 p.

12. Olson DM, Dinerstein E, Wirkramanayke ED, Burgess ND, Powll GVN, et al. (2001) Terrestrial Ecoregions of the World: A New Map of Life on Earth. BioScience 51: 933-938.

13. Hijmans RJ, Cameron SE, Parra JL, Jones PG, Jarvis A (2005) Very high resolution interpolated climate surfaces for global land areas. International Journal Of Climatology 25: 1965-1978.

14. Aschmann’s original 5th condition is that an area can have no more than 3% of the annual hours below 0°C. The WorldClim dataset and other global climatologies do not include hourly temperature data, so we were not able to map this condition. However, Aschmann does provide a proxy for this condition in his paper by using a relationship between average annual temperature and the range of average monthly temperatures. Based on the Figure 3 in his article, areas will satisfy the 5th condition if the average annual temperature is greater than 0.7 times the range in monthly temperatures plus 2.76 (8).

15. Randall DA, Wood RA, Bony S, Colman R, Fichefet T, et al. (2007) Climate Models and Their Evaluation. In: Solomon S, Qin D, Manning M, Chen Z, Marquis M et al., editors. Climate Change 2007: The Physical Science Basis Contribution of Working Group I to the Fourth Assessment Report of the Intergovernmental Panel on Climate Change. Cambridge, United Kingdom and New York, NY, USA.: Cambridge University Press. pp. 748-845.

16. Christensen J, Hulme M, Storch HV, Whetton P, Jones R, et al. (2001) Regional Climate Information – Evaluation and Projections. In: Houghton JT, Ding Y, Griggs DJ, Noguer M, Linden PJvd et al., editors. Climate Change 2001: The Scientific Basis Contribution of Working Group I to the Third Assessment Report of the Intergovernmental Panel on Climate Change. Cambridge, United Kingdom and New York, NY, USA: Cambridge University Press. pp. 583-638.

17. Christensen JH, Hewitson B, Busuioc A, Chen A, Gao X, et al. (2007) Regional Climate Projections. In: Solomon S, Qin D, Manning M, Chen Z, Marquis M et al., editors. Climate Change 2007: The Physical Science Basis Contribution of Working Group I to the Fourth Assessment Report of the Intergovernmental Panel on Climate Change. Cambridge, United Kingdom and New York, NY, USA.: Cambridge University Press. pp. 848-940.

18. Wilby RL, Charles SP, Zorita E, Timbal B, Whetton P, et al. (2004) Guidelines for use of climate scenarios developed from statistical downscaling methods. Intergovernmental Panel on Climate Change. 27 p.

19. Shongwe ME, Landman WA, Mason SJ (2006) Performance of recalibration systems for GCM forecasts for southern Africa. Int J Climatol 26: 1567-1585.

20. Benestad RE, Hanssen-Bauer I, Førland EJ (2007) An evaluation of statistical models for downscaling precipitation and their ability to capture long-term trends. Int J Climatol 27: 649-665.

21. Spak S, Holloway T, Lynn B, Goldberg R (2007) A comparison of statistical and dynamical downscaling for surface temperature in North America. J Geophys Res 112.

22. Hayhoe K, Cayan D, Field CB, Frumhoff PC, Maurerf EP, et al. (2004) Emissions pathways, climate change, and impacts on California. PNAS 101: 12422-12427.

23. Malcolm JR, Liu CR, Neilson RP, Hansen L, Hannah L (2006) Global warming and extinctions of endemic species from biodiversity hotspots. Conservation Biology 20: 538-548.

24. Mitchell TD, Carter TR, Jones PD, Hulme M, New M (2004) A comprehensive set of high-resolution grids of monthly climate for Europe and the globe: the observed record (1901–2000) and 16 scenarios (2001–2100). Tyndall Centre. 55 55.

25. Scholze M, Knorr W, Arnell NW, Prentice IC (2006) A climate-change risk analysis for world ecosystems. Proc Natl Acad Sci USA 103: 13116-13120.

26. Hannah L, Midgley G, Andelman S, Araújo M, Hughes G, et al. (2007) Protected area needs in a changing climate. Frontiers in Ecology and the Environment 5: 131-138.

27. Several other studies have created ratios of the base and future data to create precipitation anomalies in order to avoid negative precipitation values. We chose to subtract the precipitation data because the ratio anomalies approach infinity as the base precipitation falls to zero. Since we apply these ratios to finer scale current climate data, the large ratios can create erroneously large precipitation changes. Instead, we used subtraction to generate the anomalies and converted any projected negative precipitation value to zero.

28. Meehl GA, Stocker TF, Collins WD, Friedlingstein P, Gaye AT, et al. (2007) Global Climate Projections. In: Solomon S, Qin D, Manning M, Chen Z, Marquis M et al., editors. Climate Change 2007: The Physical Science Basis Contribution of Working Group I to the Fourth Assessment Report of the Intergovernmental Panel on Climate Change. Cambridge, United Kingdom and New York, NY, USA.: Cambridge University Press. pp. 590-662.
