# Supplementary material for: Climate Change, Habitat Loss, Protected Areas and the Climate Adaptation Potential of Species in Mediterranean Ecosystems Worldwide
Source: PLoS One. 2009 Jul 29;4(7):e6392. doi: 10.1371/journal.pone.0006392 (PMC2712077; doi:10.1371/journal.pone.0006392)
Supplement: Table S1 — AOGCM simulations downscaled and analyzed. Table 8.1 in Chapter 8 of the IPCC's Fourth Assessment Report contains more information about these models and the references for the ocean, atmosphere and coupling components. (0.05 MB DOC) [file pone.0006392.s002.doc]

**Table S1. AOGCM simulations downscaled and analyzed. Table 8.1 in Chapter 8 of the IPCC’s Fourth Assessment Report contains more information about these models and the references for the ocean, atmosphere and coupling components.**

| **IPCC Model Designation** | **20th century**  **(20c3m)** | **Low emissions (B1)** | **Moderate emissions (A1B)** | **High emissions (A2)** |
| --- | --- | --- | --- | --- |
| BCC-CM1 | 1 | 1 | 0 | 0 |
| BCCR-BCM2.0 | 1 | 1 | 1 | 1 |
| CCSM3 | 8 | 8 | 7 | 4 |
| CGCM3.1(T47) | 5 | 5 | 5 | 5 |
| CGCM3.1(T63) | 1 | 1 | 1 | 0 |
| CNRM-CM3 | 1 | 1 | 1 | 1 |
| CSIRO-Mk3.0 | 1 | 1 | 1 | 1 |
| ECHAM5/MPI-OM | 4 | 3 | 4 | 3 |
| ECHO-G | 3 | 3 | 3 | 3 |
| FGOALS-g1.0 | 3 | 3 | 3 | 0 |
| GFDL-CM2.0 | 1 | 1 | 1 | 1 |
| GFDL-CM2.1 | 1 | 1 | 1 | 1 |
| GISS-AOM | 2 | 2 | 2 | 0 |
| GISS-EH | 3 | 0 | 3 | 0 |
| GISS-ER | 5 | 1 | 2 | 1 |
| INM-CM3.0 | 1 | 1 | 1 | 1 |
| IPSL-CM4 | 1 | 1 | 1 | 1 |
| MIROC3.2(hires) | 1 | 1 | 1 | 0 |
| MIROC3.2(medres) | 3 | 3 | 3 | 3 |
| MRI-CGCM2.3.2 | 5 | 5 | 5 | 5 |
| PCM | 4 | 3 | 4 | 4 |
| UKMO-HadCM3 | 2 | 1 | 1 | 1 |
| UKMO-HadGEM1 | 1 | 1 | 1 | 0 |
| **Grand Total** | **58** | **48** | **52** | **36** |
