# Supplementary material for: Climate Change, Habitat Loss, Protected Areas and the Climate Adaptation Potential of Species in Mediterranean Ecosystems Worldwide
Source: PLoS One. 2009 Jul 29;4(7):e6392. doi: 10.1371/journal.pone.0006392 (PMC2712077; doi:10.1371/journal.pone.0006392)
Supplement: Table S2 — Reasons for MCE contraction for the high emissions scenario (A2) where 90–100% of the AOGCM simulations agree. Unless noted, figures are percent of current MCE in each region or for all regions. (0.04 MB DOC) [file pone.0006392.s003.doc]

**Table S2**. Reasons for MCE contraction for the high emissions scenario (A2) where 90-100% of the AOGCM simulations agree. Unless noted, figures are percent of current MCE in each region or for all regions.

| **Reason for Contraction** | **Mediterranean Basin** | **USA /**  **Mexico** | **Chile / Argentina** | **South Africa** | **Australia** | **All Regions** |
| --- | --- | --- | --- | --- | --- | --- |
| Current MCE (km^2^) | 939,904 | 148,688 | 62,408 | 32,308 | 392,408 | 1,575,716 |
| Total projected contraction | 5.4% | 3.0% | 4.8% | 15.0% | 12.6% | 7.1% |
| Too warm in winter | 1.9% | 2.3% | - | 1.8% | 10.4% | 4.0% |
| Too dry | 2.3% | - | 4.0% | 6.4% | - | 1.7% |
| Too warm in winter and too dry | 0.1% | - | - | 1.3% | - | 0.1% |
| Too much summer rain | - | - | - | 1.0% | - | 0.02% |
| <90% agreement on reason for contraction | 1.2% | 0.6% | 0.8% | 4.4% | 2.2% | 1.5% |
