# Supplementary figures and images for: Climate Change, Habitat Loss, Protected Areas and the Climate Adaptation Potential of Species in Mediterranean Ecosystems Worldwide
Source: PLoS One. 2009 Jul 29;4(7):e6392. doi: 10.1371/journal.pone.0006392 (PMC2712077; doi:10.1371/journal.pone.0006392)

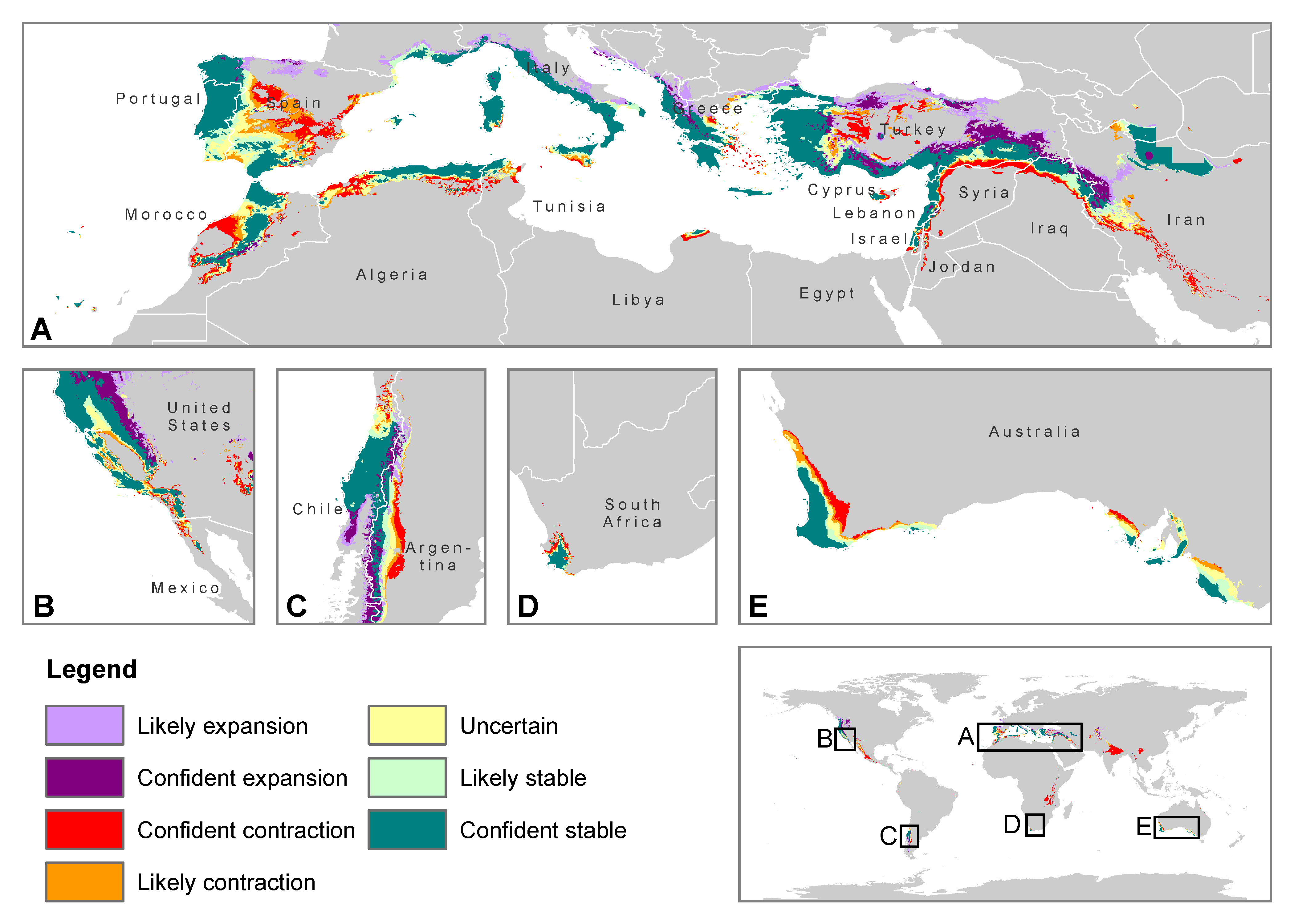

Supplement: Figure S1 — Projected status of the MCE using the Köppen definition in 2070–2099 relative to 1960–1989 under high (A2) emissions scenario. The projected status is considered likely if at least 66% of the AOGCM simulations agree, confident if at least 90% agree. Maps A. through E. are un-projected at 1∶50,000,000 scale. (0.63 MB TIF) [file pone.0006392.s004.tif]
